# The Formation of Persister Cells in Stationary-Phase Cultures of *Escherichia Coli* Is Associated with the Aggregation of Endogenous Proteins

**DOI:** 10.1371/journal.pone.0054737

**Published:** 2013-01-24

**Authors:** Daria Leszczynska, Ewelina Matuszewska, Dorota Kuczynska-Wisnik, Beata Furmanek-Blaszk, Ewa Laskowska

**Affiliations:** 1 Department of Biochemistry, Faculty of Biology, University of Gdansk, Gdansk, Poland; 2 Department of Microbiology, Faculty of Biology, University of Gdansk, Gdansk, Poland; Universitätsklinikum Hamburg-Eppendorf, Germany

## Abstract

Persister cells (persisters) are transiently tolerant to antibiotics and usually constitute a small part of bacterial populations. Persisters remain dormant but are able to re-grow after antibiotic treatment. In this study we found that the frequency of persisters correlated to the level of protein aggregates accumulated in *E. coli* stationary-phase cultures. When 3-(N-morpholino) propanesulfonic acid or an osmolyte (trehalose, betaine, glycerol or glucose) were added to the growth medium at low concentrations, proteins were prevented from aggregation and persister formation was inhibited. On the other hand, acetate or high concentrations of osmolytes enhanced protein aggregation and the generation of persisters. We demonstrated that in the *E. coli* stationary-phase cultures supplemented with MOPS or a selected osmolyte, the level of protein aggregates and persister frequency were not correlated with such physiological parameters as the extent of protein oxidation, culturability, ATP level or membrane integrity. The results described here may help to understand the mechanisms underlying persister formation.

## Introduction

All genetically homogeneous bacterial populations produce a small number of dormant cells that survive prolonged exposure to high concentrations of antibiotics [1], [2]. These multidrug- tolerant persister cells are transient phenotypic variants of wild type cells, which become sensitive to antibiotics upon re-growth. The mechanisms of persister formation are largely unknown. Numerous studies indicate that toxin-antitoxin modules are required for persistence in *E. coli*. Moyed and co-workers [3] identified in *E. coli* the first high-persistence allele *hipA7*, related to the toxin-antitoxin modules, that increased the frequency of persistence by 10 000 fold. Toxin HipA is a kinase that phosphorylates the elongation factor EF-Tu, leading to protein synthesis inhibition and cell growth arrest. It is known that the *hipA7* mutation decreases the affinity of HipA to HipB antitoxin resulting in enhanced toxicity of HipA [4]. Analysis of *E. coli* transcriptomes revealed that other toxin-antitoxin (TA) genes (*dinJ/yafQ, yefM, relBE, mazEF, ygiUT*) belong to the group of nearly 300–420 genes upregulated in persisters [5], [6]. Other genes upregulated in persisters are members of at least two functional groups: the SOS genes (*recA, umuDC, uvrAB, sulA*) and the genes of the heat and cold shock response family (*cspH*, *htrA*, *ibpAB*, *htpX*, and *clpB*). The expression of stress genes suggests that persister formation is a survival strategy, but on the basis of these results it is difficult to explain antibiotic tolerance. Lewis proposed that in persister cells antibiotics can bind to their targets but are unable to corrupt their function, because persisters are dormant and have strongly reduced translation (the target of aminoglycoside antibiotics), cell wall synthesis (the target of β-lactam antibiotics) and topoisomeraze activity (the target of fluoroquinolones) [2]. Recently, it was demonstrated that persisters have defects in reactions involved in the hydroxyl radical formation pathway [7]. Since the production of hydroxyl radicals has been proposed to be a common mechanism of cell death initiated by various antibiotics [8], it was suggested that persisters become tolerant to antibiotics by not producing hydroxyl radicals [7].

Persister formation can be decreased by deletion of the TA loci [5], [9], [10] or TA unrelated genes responsible for protein degradation (*lon*, [10]) or involved in various metabolic pathways including: *glpD* [11], *phoU* [12], *sucB* and *ubiF* [13]. However, further studies revealed that the effect of the majority of these mutations on persister frequency strongly depends on the age of the inoculum and a medium [14]. Persistence can be induced by the overexpression of various toxins: HipA [15], RelE [5], MqsR, HhA, CspD, HokA [9] and TisB [16] or proteins unrelated to the TA modules which become toxic when ectopically expressed [17]. Vázquez-Laslop *et al*. have shown that *E. coli* cells overproducing DnaJ chaperone or the *Salmonella enterica* PmrC, an enzyme that transfers phosphoethanolamine to lipid A, form 100- to 1000-fold more persisters [17]. It is possible that the toxicity of DnaJ and PmrC results from their unspecific interactions and aggregation with other cellular proteins that are essential for growth. These results and the fact that various genes coding for heat shock proteins, including *ibpAB* and *clpB,* are upregulated in persisters [5] prompted us to focus on the link between protein aggregation and persister formation. IbpAB and ClpB are molecular chaperones which participate in protein disaggregation in cooperation with DnaK. DnaK assists folding of nascent polypeptides and refolds stress-damaged proteins in reaction requiring ATP and DnaJ and GrpE co-chaperones [18]. It has been suggested that DnaK may be required for the maintenance of persister cells, as a *dnaK* deleted strain produced decreased amounts of persisters [19].

Previously, we demonstrated that during the stationary phase *E. coli* cells accumulate aggregates of misfolded proteins (multicomponent protein aggregates) and complexes of Dps *(starvation-induced protein*) with chromosomal DNA [20]. The formation of protein aggregates and insoluble Dps-DNA complexes depended on the growth conditions and was influenced by the availability of oxygen and glucose in the medium. Multicomponent aggregates contained proteins involved in a variety of cellular processes including translation, metabolism, cell architecture and stress responses. We found that in MOPS (3-N-morpholinopropanesulfonic acid) - buffered cultures the level of insoluble Dps and multicomponent protein aggregates was significantly decreased, despite the fact that *E. coli* cells entered the stationary phase at the same time as bacteria in an unbuffered medium [20]. The possible explanation of this result is that MOPS not only facilitated maintaining the pH value around 7.4 but could also accumulate in the cytoplasm as an osmolyte [21] and protect proteins against aggregation. It has been well documented that various osmotically active compounds such as glycine betaine, trehalose or glycerol, function as chemical chaperones which stabilize proteins and facilitate protein disaggregation *in vivo* and *in vitro* [22], [23]. We expected that MOPS and chemical chaperones would inhibit aggregation of proteins and influence persister formation during the stationary phase. Since persistence can be regarded as a symptom of bacterial aging [24], we also examined the influence of MOPS and osmolytes (trehalose, betaine, glucose and glycerol) on the physiological parameters that are known to change in aging cultures: the extent of protein oxidation, culturability, the level of ATP and membrane stability [25]–[27]. We asked the question whether there is a link between these physiological parameters and the frequency of persisters.

## Materials and Methods

### Growth Conditions

*E. coli* MC4100 [*araD139* Δ(*lacIPOZYA argF*) 21 *U169 fla relA rpsL*] was grown in LB medium at 37°C in Ehrlenmeyer flasks with agitation (200 rpm, aerobic cultures) or in screw-cap tubes filled to the top (anaerobic cultures). To obtain reproducible results, the medium was sterilized by filtering [14]. To prepare glycerol stocks, a culture grown to an OD_595_ = 1 was supplemented with glycerol at a final concentration of 10%, divided into 25 µl aliquots and frozen at −80°C. The 25 µl stock was used for inoculating an overnight culture (50 ml in a 250 ml flask). After 18 h the culture was diluted 1∶100 into a fresh LB medium supplemented with 40 mM MOPS pH 7.4, acetate, trehalose, betaine, glycerol or glucose at the concentration indicated in the text.

### Estimation of Persister Level

To determine the number of persisters, the cultures were diluted to an OD_595_ = 0.1 in fresh LB medium and supplemented with ampicillin (200 µg/ml). After 10 h of incubation at 37°C the surviving persisters were plated on LB agar for colony counts. To rule out the possibility of development of antibiotic resistance due to spontanic mutations persister-derived colonies were replica plated on LA plates with ampicillin. The frequency of the persisters was estimated in relation to the total number of colony forming units or total number of cells before antibiotic treatment. Total cell counts were determined using a Thoma-chamber at a 1000-fold magnification.

### Estimation of Mutation Rates

The mutation rate to amp^R^ in stationary-phase cultures (LB supplemented with 0.2% acetate, 40 mM MOPS pH 7.4, 0.2% trehalose, 0.2% betaine, 0.2% glucose or 0.2% glycerol) was determined according to [28]. In a fluctuation assay, 10 independent 5 ml cultures were tested. 1.5 ml of each stationary-phase culture was pelleted, resuspended in 150 µl of 0.85% NaCl and spread on LA plates containing ampicillin (200 µg/ml). After 48 h at 30°C, single mutant colonies were counted and the p_0_ method was used to calculate the number of mutations per culture.

### Isolation of Protein Aggregates

Cells were pelleted, converted into spheroplasts and lysed by sonication as described previously [29]. Protein aggregates were separated from *E. coli* membranes and soluble proteins according to [20]. The cell extracts were incubated with 2% Triton X-100 for 15 min at room temperature and subsequently loaded on a two step sucrose gradient (1 ml of 55%, w/w, sucrose; 5 ml of 17%, w/w, sucrose). After 1.5 h ultracentrifugation (200,000×g), a 1 ml sample containing insoluble proteins was collected from the bottom of the gradient. The insoluble proteins and whole cell extracts were resolved by SDS-PAGE [30]. The amount of aggregated protein was estimated by densitometry of the gels in relation to the total protein content in whole cell extracts (set to 100%).

### Immunodetection of Oxidized Proteins

Carbonyl groups, the major products of protein oxidation, were immunodetected after the reaction with 2,4-dinitrophenylhydrazine (DNPH). Crude protein extracts and protein aggregates were incubated with 10 mM DNPH in 2 M HCl for 30 min at room temperature. After neutralization with 2 M NaOH, the proteins were dissolved in Laemmli lysis buffer, separated by SDS-PAGE and transferred to a nitrocellulose membrane [30]. Protein-bound 2,4-dinitrophenylhydrazones were visualized using anti-2,4-dinitrophenol (DNP) antibodies (Sigma) and ECL Western blotting detection reagents (Roche).

### Determination of the Relative ATP Levels

100 µl of bacterial culture was mixed with an equal volume of the BacTiter-Glo reagent (Promega) prepared according to the manufacturer’s instructions. The sample was mixed thoroughly and incubated at room temperature for 30 s. The luminescence of the sample was measured using a luminometer (Junior, EG&G Berthold).

### Determination of Membrane Integrity

1 ml of culture was pelleted, washed and resuspended in 1 ml of 0.9% NaCl. The sample was stained with 3 µl of a mixture containing 0.33 mM SYTO9 and 2 mM propidium iodide (LIVE/DEAD BacLight viability kit, Molecular Probes). After 15 min incubation at room temperature in the dark the fluorescence emission spectrum (excitation at 470 nm, emission at 490–700 nm) was measured using a Perkin-Elmer LS-5B spectrofluorimeter. The ratio of integrated green fluorescence (510–540 nm) to integrated red fluorescence (620–650 nm) was calculated according the manufacturer’s instructions.

## Results

### The Frequency of Persisters is Associated with the Level of Protein Aggregates but not with the Amount of Oxidized Proteins Accumulated in Stationary-phase *E. coli* Cultures

**Figure 1:**
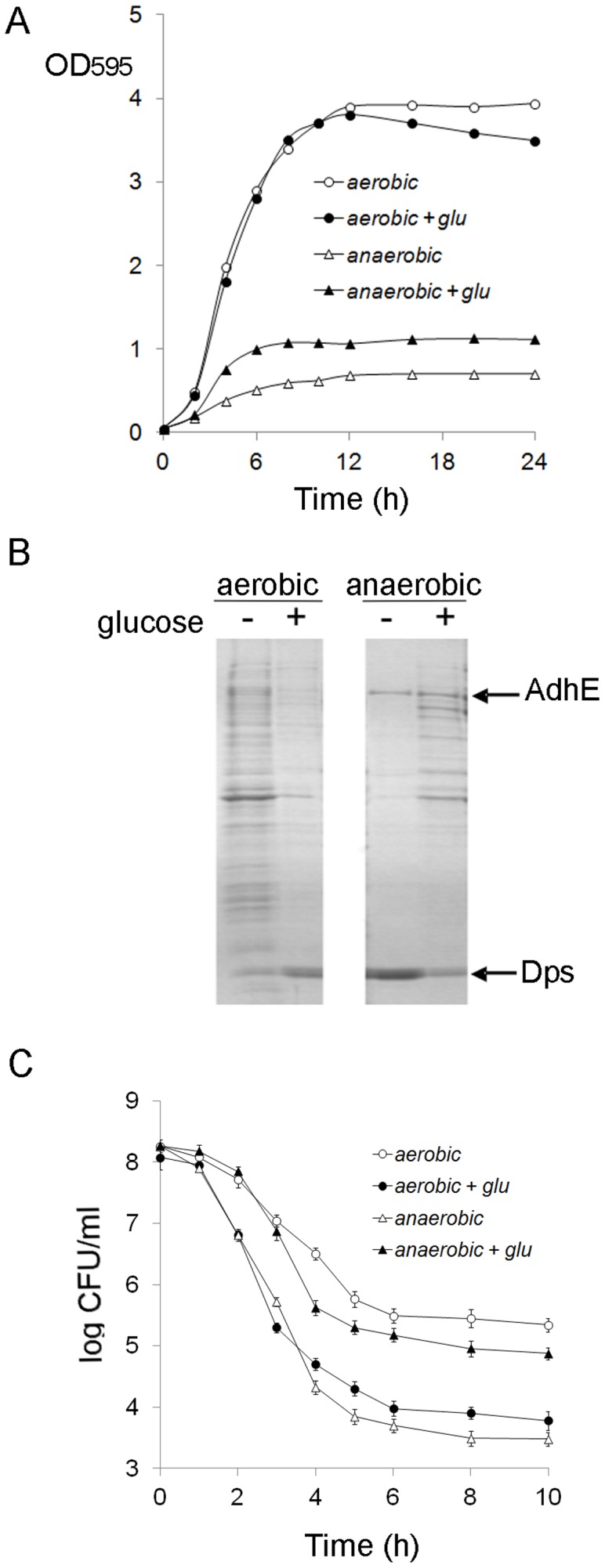
Stationary-phase *E. coli* cultures that accumulate protein aggregates produce increased levels of persisters. (A) Growth curves of *E. coli* MC4100. The bacteria were grown aerobically (circles) or anaerobically (triangles) at 37°C in LB medium supplemented (black symbols) or not (white symbols) with 0.2% glucose. (B) Protein aggregates were isolated from the stationary-phase cultures as described in Materials and Methods. Equal volumes of insoluble fractions isolated in the sucrose gradient were resolved by SDS-PAGE and visualized by Coomassie staining. (C) After 24 h the cultures were diluted to an OD_595_ = 0.1 and exposed to ampicillin (200 µg/ml) for 10 h at 37°C. Antibiotic- tolerant bacteria were plated for colony counting at the times indicated in the graph. The error bars indicate the standard deviations of three independent experiments.

Protein aggregation and persister formation were investigated in 24 h stationary *E. coli* cultures (Figure 1A). Previously, we had found that the formation of multicomponent protein aggregates and insoluble Dps-DNA complexes, depended on growth conditions and was influenced by the availability of oxygen and glucose in the medium [20]. Insoluble protein fraction isolated from aerobic stationary cells grown in LB medium contained multicomponent protein aggregates whereas the aggregates from the culture supplemented with 0.2% glucose contained Dps as almost a sole component (Figure 1B). On the contrary, under oxygen depletion, multicomponent protein aggregates appeared in the presence of glucose, whereas a high level of insoluble Dps and an additional protein AdhE were detected in the absence of glucose. Multicomponent protein aggregates in aerobic (LB) and anerobic (LB+glu) cultures constituted ∼ 4±0.5% and 2±0.5% of total cellular proteins, respectively. Treatment of the cultures with ampicillin resulted in biphasic killing kinetics with an initial rapid death for the bulk of the populations followed by a slower killing rate for the persisters (Figure 1C). All the tested cultures differed in the frequency of persisters. The highest level of persisters after ampicillin treatment was detected in the aerobic culture (LB) which contained a high level of protein aggregates. The addition of 0.2% glucose to LB medium inhibited protein aggregation (Figure 1B) and accelerated cell lysis in the presence of ampicillin (Figure 1C). Under anaerobic conditions, cell death was more efficient in the absence of glucose resulting in an approximately 15-fold decrease of persisters in comparison to the anaerobic culture with glucose (Figure 1C). These results suggested that the increased frequency of persisters during the stationary phase is specific for conditions that promote formation of multicomponent protein aggregates but not insoluble Dps-DNA complexes.

**Figure 2:**
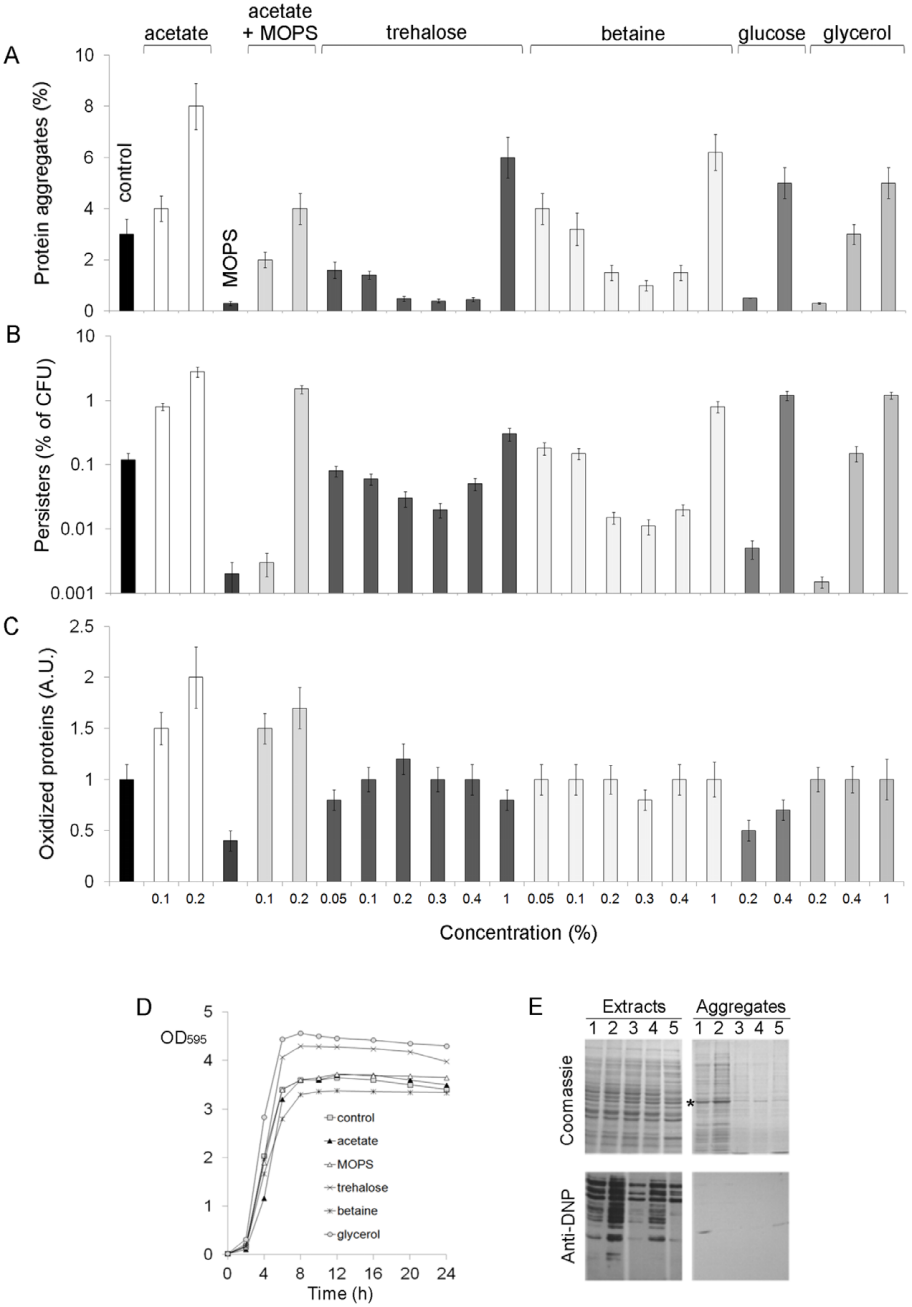
Aggregation of proteins, formation of persisters and oxidation of proteins in the presence of acetate, MOPS and osmolytes. Bacteria were grown aerobically for 24 h at 37°C in LB medium supplemented with 0.2% acetate, 40 mM MOPS pH 7.4, 0.2% glucose, 0.2% glycerol, 0.2% trehalose or 0.2% betaine, unless otherwise stated. The control culture was grown in LB without supplements. Protein aggregates were isolated from 24 h cultures, resolved by SDS-PAGE and visualized by Coomassie staining. Oxidized, DNPH-derivatized proteins were resolved by SDS-PAGE and immunodetected using anti-dinitrophenol antibodies (E). The amount of aggregated proteins in relation to total protein in cell extracts (A) and the relative level of oxidized proteins (C) were calculated on the basis of densitometry. (B) After 24 h the cultures were diluted to an OD_595_ = 0.1 and exposed to ampicillin (200 µg/ml) for 10 h at 37°C. Antibiotic- tolerant bacteria were plated for colony counting at the times indicated in the graph. 100% corresponds to the number of colonies before antibiotic treatment. Means and standard deviation of three independent experiments are shown. (D) Representative growth curves of selected cultures are shown. (E) Samples corresponding to the same OD_595_ were loaded on the gels. Lane 1- the control culture, lane 2- LB+acetate, lane 3 - LB +MOPS, lane 4 - LB+trehalose, lane 5- LB+glucose. The asterics indicates EF-Tu, the most abundant protein in the aggregates.

To further confirm that the frequency of persisters is associated with the level of protein aggregates, we analyzed the influence of different carbon sources on protein aggregation and persister formation in aerobic stationary cultures. We found that 0.2% lactose, mannose or galactose did not significantly influence either the formation of multicomponent protein aggregates or the frequency of persisters (data not shown). On the contrary, when LB medium was supplemented with 0.2% sodium acetate the bacteria accumulated high amounts of protein aggregates (7–9% of total cellular proteins) and produced increased levels of persisters (Figure 2A, B). Buffering the medium with 40 mM MOPS pH 7.4 resulted in a decreased protein aggregation, as previously described [20], and reduced persister level in the control (LB with no supplements) and acetate-supplemented cultures. A similar effect was observed in the presence of 0.05–0.4% trehalose or 0.2–0.4% betaine, however a higher concentration (1%) of these osmolytes stimulated protein aggregation as well as the formation of persisters. Increased persister and aggregate levels were also found in cultures with 0.4% glucose or 1% glycerol, but lower concentrations of glucose (0.2%, Figure 2A, B) and glycerol (0.2%, Figure 2A, B) significantly inhibited both protein aggregation and persister formation. It should be noted that biphasic killing kinetics in the cultures with the supplements was observed (data not shown) confirming the presence of persisters. Although 0.2% trehalose or 0.2% glycerol stimulated *E. coli* growth, all the tested cultures entered the stationary phase approximately at the same time as the control culture, e. g. between the 8^th^ and 10^th^ h (Figure 2D). To rule out the possibility that isolated persisters were resistant mutants, and the supplements in the medium affected mutation rates, persister colonies were replica plated on LA plates with ampicillin. No ampicillin resistant colonies were detected. In addition, we determined the mutation rate to amp^R^ in the stationary phase cultures according to Rosche and Foster [28]. The average spontaneous mutation rate of *E. coli* MC4100 to resistance to 200 µg/ml ampicillin in stationary-phase control culture (LB without supplements) was found to be ∼3×10^−11^ mutations per cell division. The mutation rate was not influenced by the presence of 0.2% acetate, 40 mM MOPS pH 7.4 or the osmolytes.

**Figure 3:**
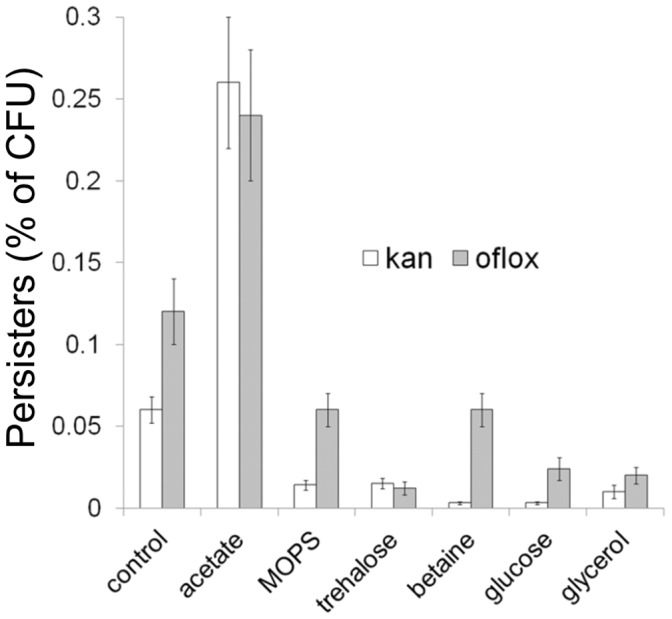
The effect of acetate, MOPS and osmolytes on the frequency of persister cells tolerant to kanamycin and ofloxacin. Bacteria were grown aerobically for 24 h at 37°C in LB medium supplemented with 0.2% acetate, 40 mM MOPS pH 7.4, 0.2% glucose, 0.2% glycerol, 0.2% trehalose or 0.2% betaine. The control culture was grown in LB without supplements. To isolate persisters the cultures were diluted to an OD_595_ = 0.1 and exposed to kanamycin (20 µg/ml) for 4 h or to ofloxacin (5 µg/ml) for 6 h at 37°C. The frequency of persisters was estimated as described in the legend to Figure 2. Means and standard deviation of three independent experiments are shown.

Taken together, these results showed that more persisters were formed when the aggregation of proteins in the stationary-phase cells was enhanced. In parallel, the inhibition of protein aggregation prevented formation of persisters. We confirmed that these effects also can be observed when persisters were isolated after exposure of the culture to aminoglicoside kanamycin and fluoroquinolone ofloxacin. Acetate increased the level of cells persistent to kanamycin and ofloxacin, whereas, MOPS and osmolytes decreased the level of persisters tolerant to kanamycin and ofloxacin (Figure 3).

Aggregation of proteins is one of the damages observed in aging *E. coli* cultures [20], [31]. Another hallmark of bacterial senescence is protein oxidation [25]. It was demonstrated that oxidative stress can increase persistance [32] and persisters have been proposed as aging bacteria [24]. Therefore, it seemed possible that MOPS and osmolytes not only inhibited protein aggregation (Figure 2A) but also protected proteins against oxidation and thus postponed the aging of stationary-phase cells and the generation of persisters. However, we found that there was no simple relationship between persistence and protein oxidation (Figure 2B, C). Trehalose, betaine and glycerol, at a concentration that inhibited or promoted protein aggregation and persister formation did not significantly affect the level of oxidized proteins. Protein oxidation was decreased in the presence of MOPS and glucose, and increased in cells exposed to acetate. It is worth noting that oxidized proteins remained soluble and were hardly detectable in the aggregates (Figure 2E). Taken together, these results showed that the frequency of persisters in stationary *E. coli* cultures correlates with the level of protein aggregates but is independent of the amount of oxidized proteins.

### The Tendency to form Persisters in *E. coli* Stationary-phase Cultures does not Correlate with Culturability, the Level of ATP or Membrane Stability

**Figure 4:**
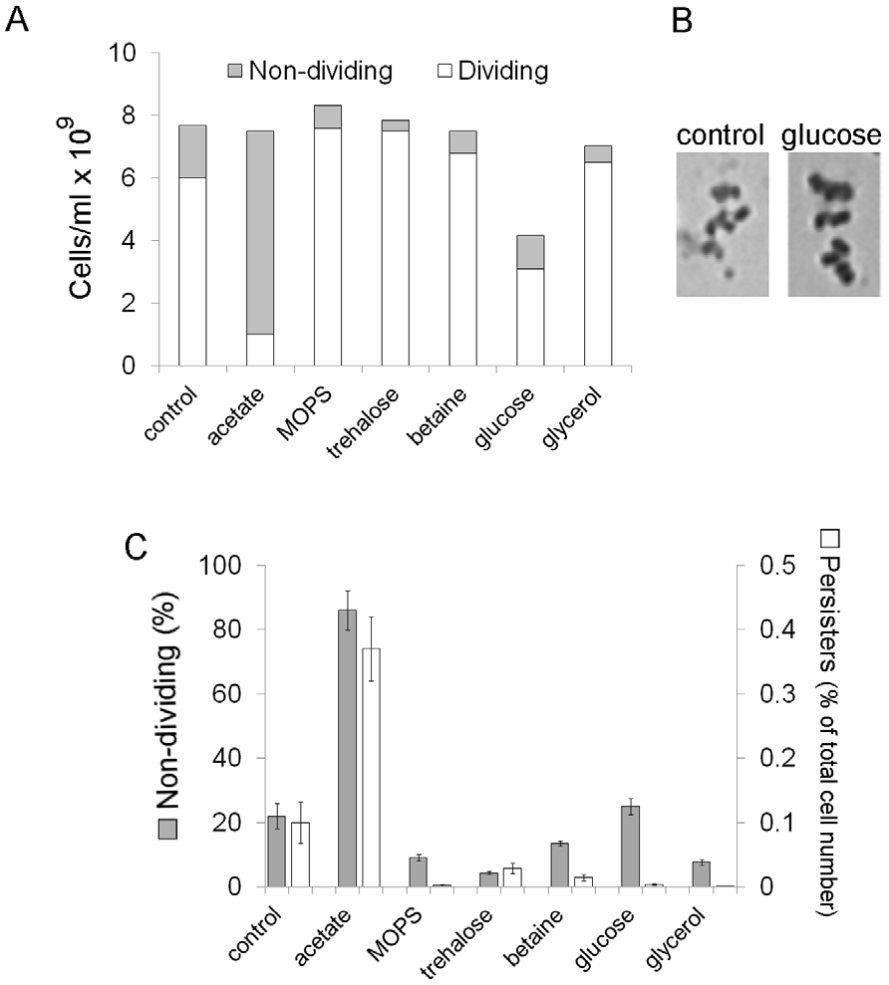
The influence of acetate, MOPS and osmolytes on the levels of non-dividing and persister cells. Bacteria were grown aerobically for 24 h at 37°C in LB medium supplemented with 0.2% acetate, 40 mM MOPS pH 7.4, 0.2% glucose, 0.2% glycerol, 0.2% trehalose or 0.2% betaine. The control culture was grown in LB without supplements. (A) The total number of cells per ml was obtained using a hemocytometer. Dividing cell counts were estimated by plating serial dilutions on LA. The number of non-dividing cells was calculated by subtracting colony-forming units counts from the total number of cells. (B) *E. coli* stationary- phase cells grown in the presence of glucose are larger than control cells. 1000×magnification. (C) To isolate and estimate persisters the stationary cultures were diluted to an OD_595_ = 0.1 and exposed to ampicillin (200 µg/ml) for 10 h at 37°C. Antibiotic- tolerant bacteria were plated for colony counting. 100% corresponds to the total number of cells (dividing and non-dividing) before antibiotic treatment. Means and standard deviation of three independent experiments are shown.

To obtain a more accurate insight into the mechanism of persister cell formation we studied the effect of acetate, MOPS and osmolytes on the culturability, ATP level and membrane stability in *E. coli* stationary –phase cultures. In these experiments, the frequency of persisters was expressed as a percentage of total cell number (Figure 4) unlike in Figure 2B, where persister level was presented as a percentage of CFU. Regardless of how the persister fraction was estimated, we observed similar effects (compare Figures 2B and 4C): increased (0.2% acetate) and decreased (MOPS, 0.2% trehalose, 0.2% betaine, 0.2% glucose or 0.2% glycerol) persister levels. We found that almost 90% of the acetate stressed cells, which generated the highest number of persisters, were unable to divide and form colonies, whereas in the presence of MOPS, 0.2% trehalose, 0.2% betaine or 0.2% glycerol, which inhibited formation of persisters, the level of non-dividing cells significantly decreased (Figure 4A, C). These results suggested that the frequency of persisters was roughly proportional to the number of non-dividing cells. However, we found that 0.2% glucose did not influence the level of non-dividing cells (25% of total cell number in both the control and glucose supplemented cultures, Figure 4C), despite the fact that the frequency of persisters formed in the presence of 0.2% glucose was very low. Therefore, culturability (the number of dividing and colony forming cells) is not a proper factor that can be used to predict the tendency of bacterial cultures to form persisters. We also observed that the cells grown in the presence of glucose were approximately 2-fold larger than the control cells from LB medium without supplements (Figure 4B). Nevertheless, both cultures reached a comparable OD_595_ (Figure 1A).

**Figure 5:**
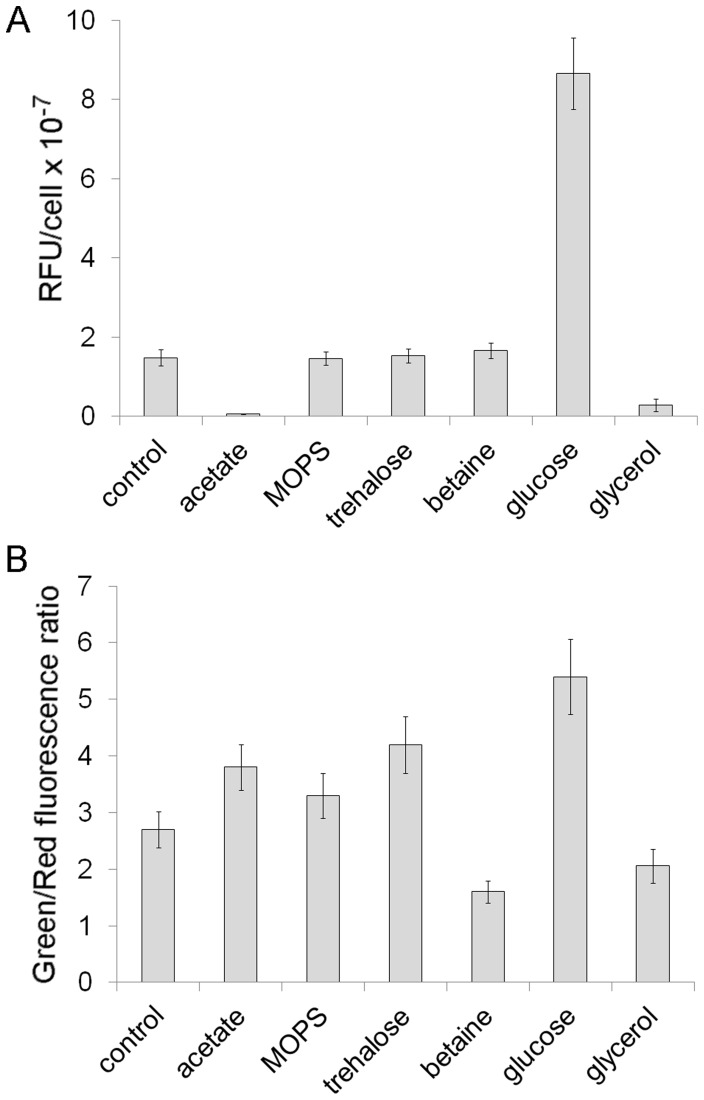
The level of ATP and membrane stability in *E. coli* stationary-phase cultures. The cultures were grown as described in the legend to Figue 4. (A) ATP level was determined using BacTiter Glo chemicals (Promega). (B) Membrane stability was assessed after staining cells with the LIVE/DEAD assay solution (Invitrogen). Error bars represent standard deviation of three independent experiments. RFU, relative fluorescence units.

We expected that the stationary cultures that generated lower levels of persisters were metabolically more active, e.g. they would contain substantially more ATP than the cultures with high persister frequencies. According to this presumption, we detected the highest and the lowest level of ATP in the culture supplemented with glucose and in acetate- stressed cells, respectively. However, in the other cultures, decreased frequency of persisters did not correlate with a higher concentration of ATP - glycerol reduced the level of ATP, whereas MOPS, trehalose and betaine did not affect ATP levels (Figure 5A).

To investigate membrane stability we used a mixture of the SYTO 9 and propidium iodide stains (LIVE/DEAD BacLight Bacterial Viability Kit ) and measured the green to red fluorescence ratio (Figure 5B). SYTO 9 stains live and damaged cells, whereas propidium iodide does not stain intact bacteria but enters cells that have lost their membrane integrity. Therefore, membrane damage should result in a decreased green to red fluorescence ratio. We found that betaine and glycerol added to LB medium noticeably decreased the green/red fluorescence ratio, indicating that more cells with damaged membranes were present in these cultures when compared to the control. Unexpectedly, acetate caused the opposite effect. Although acetate-stressed cells accumulated high levels of aggregated proteins and oxidatively damaged proteins (Figure 2), and most of them were unable to form colonies (Figure 4A), we did not observe any substantial loss of their membrane integrity. The green/red fluorescence ratio measured in bacteria from the acetate supplemented culture was even higher than in the control cells (Figure 5B). A beneficial effect on membrane integrity was also observed in the cultures supplemented with trehalose and glucose. These results indicate that MOPS and osmolytes which inhibited both protein aggregation and persister formation differentially affected cell culturability, ATP level and membrane stability.

### Antibiotic Treatment of Persisters can be more Effective in the Presence of MOPS and Osmolytes

Since MOPS and osmolytes exerted beneficial effects on the various physiological parameters of *E. coli* cultures, we asked the question whether they could also help dormant persisters to “wake up” and start to divide, thereby making them sensitive to ampicillin. If this were the case, the frequency of persisters isolated after the dilution of the stationary-phase cultures into a fresh medium (LB+amp) supplemented with MOPS or a selected osmolyte should be decreased. However, such an effect was not observed (data not shown). This indicates that although MOPS and osmolytes prevent the formation of persisters in stationary phase -cultures, they do not enhance resuscitation of dormant persisters during subsequent ampicillin treatment.

**Figure 6:**
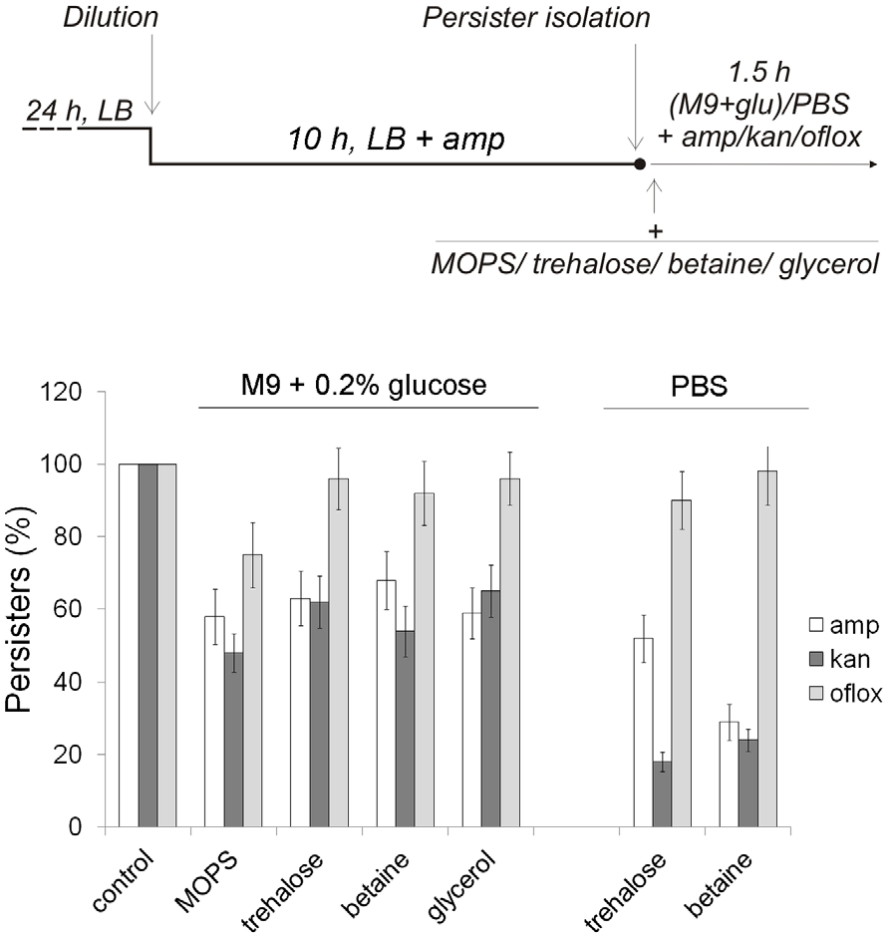
Persisters killing by antibiotics is more efficient in the presence of osmolytes. To isolate persisters, 24 h stationary culture (LB) was diluted to an OD_595_ = 0.1 in a fresh LB medium supplemented with amp (200 µg/ml). After 10 h of incubation at 37°C, persisters were pelleted, washed with 0.9% NaCl, resuspended in either M9 medium with 0.2% glucose or PBS. Persisters were then exposed to antibiotics (200 µg/ml amp, 20 µg/ml kan, 3 µg/ml oflox) at 37°C for 1.5 h in the absence or in the presence of 40 mM MOPS pH 7.4, 0.2% trehalose, 0.2% betaine or 0.2% glycerol. Means and standard deviation of three independent experiments are shown.

Further studies carried by us revealed that under certain conditions MOPS and osmolytes may cause reversion of persisters to antibiotic-sensitive cells. Allison *et al.* demonstrated that specific metabolites, including glucose and other sugars that enter upper glycolysis, do not cause re-growth of persisters in M9 minimal medium but stimulate killing of persisters with aminoglycosides by inducing proton motive force which facilitates aminoglycosides uptake [33]. We found that MOPS and osmolytes further enhanced this effect (Figure 6). In our experiment, persisters were isolated after ampicillin treatment for 10 h, washed and resuspended in M9 minimal medium supplemented with 0.2% glucose and subjected again to antibiotic treatment in the presence of MOPS or selected osmolytes. In accordance with [33], we found that glucose sensitized persisters to aminoglycoside kanamycin (data not shown). When MOPS, trehalose, betaine or glycerol were added to the medium an additional 30–65% of persisters were killed by kanamycin. Interestingly, in the presence of MOPS or the osmolytes 35–40% of the persisters also became susceptible to ampicillin. In addition, MOPS partly sensitized persisters to ofloxacin. We also found that trehalose- and betaine- dependent sensitization of persisters to ampicillin and kanamycin was more effective in phosphate-saline buffer (PBS, pH 7.2) than in M9 medium. In PBS supplemented with trehalose or betaine, 50% and 70% of the persisters, respectively, were killed by ampicillin treatment, and 80% of the persisters were killed by kanamycin (Figure 6).

## Discussion

Multiple mechanisms that control the formation of persisters are not yet fully understood. In this study we demonstrated that increased production of persisters is correlated with aggregation of proteins in *E. coli* stationary phase cultures. We manipulated the level of protein aggregates by supplementing the media with acetate, MOPS or various osmolytes (trehalose, betaine, glycerol and glucose). Acetate increased the protein aggregation and frequency of persisters, whereas MOPS and osmolytes inhibited the aggregation of proteins and generation of persisters.

The mechanism or mechanisms that link protein aggregation and persister formation remain unclear, however, at least two hypothesis can be proposed to explain this association. There is increasing evidence that protein aggregates are not distributed evenly within bacterial population [34], [35]. Protein aggregates were found to be mainly deposited at the older polar sites and inherited after division by only one progeny cell. It is possible that bacteria that accumulate protein aggregates faster than other cells (due to asymmetrical inheritance of the aggregates or in a stochastic way) become persisters. The sequestering of vital proteins into aggregates may slow down their metabolism leading to a dormant state. We found that the main component of the aggregates was the elongation factor EF-Tu [20], one of the most abundant *E. coli* proteins. EF-Tu was overrepresented in the aggregates (Figure 2E) indicating that it is prone to aggregation upon stationary phase. The aggregation of EF-Tu may lead to the inhibition of translation resulting in the generation of dormant, persistent cells. This presumption is in agreement with earlier studies demonstrating that HipA toxin is implicated in persister formation trough phosphorylation – dependent inactivation of EF-Tu [4]. The second possible scenario that links protein aggregation and persistency concerns protease Lon, which was recently demonstrated to be required for persistence. Lon by degradation of various antitoxins, activates toxic endonucleases that degrade mRNAs [10]. Thus, two processes may lead to the inhibition of translation and dormancy - the inactivation of the elongation factor EF-Tu and degradation of mRNAs. Lon is also involved in degradation of unfolded and aggregated proteins [36]. It seems that the decision as to whether the cell becomes persistent or not may depends on the prevalence of one of the two Lon-mediated pathways – degradation of protein aggregates (in non-persistent cells) or degradation of antitoxins (in persistent cells). According to this hypothesis, in a cell in which Lon is engaged mainly in the removal of protein aggregates, antitoxins are relatively stable and persistence is not induced; when Lon mainly degrades antitoxins, the frequency of persisters increases and protein aggregates remain stable. We attempted to compare levels of protein aggregates in persisters and antibiotic-sensitive cells and we found that the amount of protein aggregates was 5–10 times higher in persisters than those isolated from the whole bacterial population (antibiotic-sensitive cells and persisters). However, interpretation of these results is difficult due to limitations of the method used for persister isolation [2]. It should be noted that protein aggregation in persisters can occur not only during the stationary phase but also upon exposure of the cultures to ampicillin. To avoid antibiotic treatment during persister isolation, flow cytometry can be applied as described in [6]. Unfortunately, this method was unsuitable in our studies, since we found that the dilution of the stationary cultures into a buffer (a necessary step during cell sorting) might influence the level of protein aggregates.

We demonstrated that in the presence of 40 mM MOPS pH 7.4, or the osmolytes (0.05–0.4% trehalose, 0.2–0.4% betaine, 0.2% glucose or 0.2% glycerol) aggregation of proteins in the stationary phase was inhibited (Figure 2A). Various mechanisms can be responsible for this effect. The main carbon sources in LB are peptides and amino acids; therefore, under aerobic conditions, cells release an excess of amine-containing compounds, causing alkalization of the culture (pH 9) at the stationary phase [37]. *E. coli* can adapt to external pH (in a range between 5.0 and 9.0) and maintain constant intracellular pH (∼ 7.0) by mechanisms involving pH-dependent catabolism and ion fluxes [38]. Therefore, it seems that proteins which aggregated in *E. coli* stationary-phase cells were not directly exposed to an alkaline environment. Nevertheless, in cultures buffered with 40 mM MOPS, the pH of the medium was maintained at 7.4 until the end of the experiment [20] and protein aggregation was significantly decreased (Figure 2A). Kitko *et al*. found that diverse osmolytes including NaCl, KCl, proline or sucrose contribute to cytoplasmic pH homeostasis [38]. It is not excluded that the osmolytes used in our work caused similar effects. The results presented by Kitko [38] and other studies indicate that osmolytes can protect cells in various ways other than osmotically. Osmolytes may act as antioxidants, control redox balance, serve as energy and carbon reserve, and stabilize membranes and proteins (reviewed in [38], [39]). Osmolytes may act as chemical chaperones; betaine, proline, trehalose and glycerol have been demonstrated to protect proteins from heat denaturation and facilitate the formation of native oligomers *in vitro* and *in vivo* [22], [23], [40]–[43]. Chemical chaperones may indirectly enhance the efficiency of protein renaturation by stabilizing molecular chaperones that participate in the refolding reaction [22], [23]. The protective effect of various osmolytes can be observed when their production is induced in the cell in response to changes in the environment or when osmolytes are added externally to growth media. In *E. coli,* for example, the viability of a *dnaK* deletion mutant was restored at a higher temperature in the medium supplemented with glycine betaine or choline [41], while externally added betaine or K-glutamate enabled protein disaggregation *in vivo* in wild type cells at high temperatures [23]. We suppose that the inhibition of protein aggregation in the stationary phase was caused by various processes associated with changes in the metabolism during the adaptation to osmolytes as carbon sources, pH homeostasis, the activity of osmolytes as chemical chaperones and osmolytical activity. Similar mechanisms could be responsible for the partial reversion of persisters to antibiotic sensitive cells (Figure 6). The exact mechanisms underlying these processes remain to be elucidated.

We found that higher concentrations of trehalose, betaine, glucose and glycerol had a contrary effect comparing to the moderate concentrations, and stimulated production of persisters and protein aggregates. These results can be explained by findings indicating that higher concentrations of some osmolytes may destabilize and unfold [44] or “overstabilize” proteins [40]. It is proposed that overstabilized proteins can acquire rigid conformation and tend to precipitate. We also suppose that high concentrations of glucose in the medium led to the overproduction of acetate [37] which in turn caused a similar effect as the supplementation of the medium with sodium acetate. The high frequency of persisters detected in acetate stressed cultures (Figure 2B) is in agreement with results published by Hong and co-workers [32] which indicated that persisters are generated in response to external stresses, including acid stress (pH 2.5).

Our studies revealed that protein aggregation and persister frequency were not correlated with the oxidation of proteins. Proteins oxidized upon the stationary phase were almost exclusively detected in a fraction of soluble proteins (Figure 2E). This result was not consistent with data published by Maisonneuve *et al.* [31], which demonstrated that carbonylated proteins formed aggregates upon the stationary phase. This discrepancy can be explained on the basis of results described by Spiegeleer *et al.* [45]. The authors observed significant differences in the level of oxidized proteins in bacteria, depending on the source of tryptone, which was a component of the LB medium. They concluded that cells may experience different subinhibitory levels of oxidative stress in tryptone-containing growth media. In some cases this may trigger a stress response that protects proteins against subsequent oxidation in the stationary phase. It seems that such a situation might happen under the conditions used in our experiments. In consequence, the extent of protein oxidation might be too low to promote formation of the aggregates [46].

In the stationary phase bacteria gradually lose their culturability and non-dividing cells are unable to retrieve growth even when nutrients become available again [25]. We found that MOPS and osmolytes decreased both the frequency of persisters and the number of non-dividing cells. However, the culture supplemented with glucose was an exception. Glucose significantly inhibited the generation of persisters but did not influence the level of non-dividing cells. Therefore, culturability may not necessarily reflect the tendency of bacterial cultures to form persisters. We also found that the size of bacteria grown in the presence of glucose was increased ∼2-fold (Figure 4B). Both glucose-supplemented and control cultures reached approximately the same OD_595_ (Figure 1A). Since total cell volume in a sample correlates with culture OD [47], we assumed that total cell volume and biomass were comparable in both cultures. Numerous data indicates that cell size is directly correlated with nutrient source and growth rate. Recently, UDP-glucose has been proposed as a universal proxy for nutrient availability [48]. During growth in rich media high intracellular concentrations of UDP-glucose affect assembly of the FtsZ ring leading to the inhibition of cell division and increasing cell size. During starvation the concentration of UDP-glucose is low, hence cell divisions are permitted resulting in reduced cell size. We suppose that the size of cells grown in the presence of glucose was increased (Figure 4B) due to a higher intracellular concentration of UDP-glucose.

A low ATP level is also a typical feature of dormant cells and aging bacterial cultures [27]. Therefore, we expected that the level of ATP would be considerably reduced in cultures with increased frequencies of persisters. On the other hand, ATP level should be relatively higher in the cultures that showed diminished persister levels. However, our results revealed that there is no simple relationship between the levels of ATP and persisters (Figures 2B and 5A). It should be kept in mind that ATP levels were determined in bulk populations. It is possible that the cell could become persistent when its ATP level drops below a certain threshold. It is not known whether ATP was distributed evenly or not in the rest of the population. For example, a culture with an increased frequency of persisters may contain a subpopulation of non-persistent cells producing high amounts of ATP, whereas a subpopulation of bacteria with a relatively low ATP content may be present in a culture characterized by low persister frequency. In consequence, the overall ATP level estimated in both cultures could be comparable.

We did not observe any correlation between persister frequency and membrane integrity. Interestingly, in acetate stressed cells which were characterized by low culturability (Figure 4A) and a very low ATP level (Figure 5A) membrane integrity measured with the LIVE/DEAD viability kit was not affected (Figure 5B). This may suggest that acetate induced formation of VBNC (viable-but-not-culturable) cells which retain their viability although are metabolically inactive and have lost culturability [49]. The results presented in Figure 5 point to the fact that depending on the criterion for viability (ATP level or membrane integrity), contradictory conclusions can be drawn.

In summary, our findings demonstrate that there is a strong correlation between accumulation of protein aggregates in the cell and generation of persisters. During persister formation protein misfolding and aggregation may occur as one of the crucial steps that induce dormancy. We found that the frequency of persisters is independent of the physiological parameters: the extent of protein oxidation, culturability, the level of ATP and membrane integrity. This information may help to establish new strategies to prevent the formation of persisters, which are the main cause of the recalcitrance of bacterial infections to antimicrobial agents.
